# Stopping antibiotic therapy after 72 h in patients with febrile neutropenia following intensive chemotherapy for AML/MDS (safe study): A retrospective comparative cohort study

**DOI:** 10.1016/j.eclinm.2021.100855

**Published:** 2021-04-25

**Authors:** A. Schauwvlieghe, A. Dunbar, E. Storme, A. Vlak, R. Aerts, J. Maertens, B. Sciot, T. Van Der Wel, G. Papageorgiou, I. Moors, J.J. Cornelissen, B.J.A. Rijnders, T. Mercier

**Affiliations:** a Department of hematology, Ghent University Hospital, Gent, Belgium; b Internal Medicine, Infectious Diseases, Erasmus University Medical Center, Rotterdam, Netherlands; c Department of Hematology, Universitaire Ziekenhuizen Leuven, KU Leuven, Belgium; d Department of Biostatistics, Erasmus University Medical Centre, Rotterdam, Netherlands; e Department of Hematology, Erasmus MC Cancer Institute, Erasmus University Medical Center, Rotterdam, Netherlands; f Department of Microbiology, Immunology and Transplantation, Katholieke Universiteit Leuven, Leuven 3000, Belgium

**Keywords:** Febrile neutropenia, Leukaemia, Opportunistic infections, Antimicrobial stewardship, Multi-drug resistant bacteria, Microbiome

## Abstract

**Background:**

Induction chemotherapy for acute myeloid leukemia (AML) or myelodysplastic syndrome (MDS) is almost universally complicated by febrile neutropenia(FN). Empirical broad-spectrum antibiotic therapy (EBAT) strategies advocated by guidelines result in long periods of broad-spectrum antibiotic therapy. We compared the outcome of AML/MDS patients treated with a 3-day versus a prolonged (until neutrophil recovery) regimen.

**Methods:**

This is a retrospective comparative cohort study in AML or MDS patients undergoing remission-induction chemotherapy from 2011 to 2019, comparing 2 tertiary care hospitals with different strategies regarding antibiotic treatment for FN. At Erasmus University medical center(EMC), EBAT was stopped after 3 days of FN, in absence of a clinically or microbiologically documented infection. In the University Hospitals Leuven(UZL), a prolonged strategy was used, where EBAT was given until neutrophil recovery. The primary endpoint was a serious medical complication(SMC) defined as death or ICU admission in the 30 days after the start of chemotherapy.

**Findings:**

305 and 270 AML or MDS patients received chemotherapy at EMC and UZL, respectively. Broad-spectrum antibiotic treatment was given for a median of 19 days (IQR13-25) at UZL versus 9 days at EMC (IQR5–13) (*p* <0·001). With the 3-day EBAT strategy, an SMC was observed in 12·5% versus 8·9% with the prolonged strategy (*p* = 0·17). The hazard ratio for an SMC was not significantly higher with the 3-day strategy (HR 1·357,95%CI 0·765–2·409).

**Interpretation:**

This study suggests that during remission induction chemotherapy it is safe to stop antibiotics after 3 days of FN in absence of infection. A comparison of both strategies in a prospective trial should be pursued.

## 1 Introduction

Patients with Acute Myeloid Leukemia (AML) or myelodysplastic syndrome (MDS), treated with classic “3 + 7″ induction chemotherapy, always go through an episode of longstanding neutropenia of three to four weeks. In more than 80% of these patients, at least one episode of febrile neutropenia (FN) is observed [1]. As fever might be the first sign of an overwhelming infection, all patients with FN receive empirical broad-spectrum antibiotic treatment (EBAT). However, it is unclear for how long EBAT should be continued. The current guideline of the Infectious Diseases Society of America (IDSA) recommends to continue treatment with broad-spectrum antibiotics until bone marrow recovery [1]. The European Conference of Infections in Leukemia (ECIL-4) guidelines states that antibiotics can be discontinued after 72 h if the patient is hemodynamically stable and has been afebrile for 48 h, irrespective of neutrophil count and expected duration of neutropenia [2]. These strategies result in long periods of broad-spectrum antibiotic use, although an infectious cause can typically be documented in 20–30% of patients with FN [1]. To reduce side effects, antibiotic selective pressure on the human microbiota, and antimicrobial resistance development, a more rational use of antibiotics is necessary [3]. Therefore, if safe, stopping antibiotic therapy earlier in patients without signs of a bacterial infection could provide significant benefits.

**Research in context**

**Evidence before this study**

Current strategies towards febrile neutropenia result in long periods of broad-spectrum antibiotics usage with its associated adverse effects, even though an infectious cause can be found in only 20–30% of patients. Only one randomized controlled trial suggests that discontinuing broad-spectrum antibiotics after 72 h of apyrexia is safe and can result in significantly fewer days of broad-spectrum antibiotic use.

**Added value of this study**

The policy we investigated here goes one step further because broad-spectrum antibiotics are also being stopped after 72 h, even when fever has not resolved. One single center prospective observational study evaluated the same approach but cannot be considered sufficient evidence as a comparative group was lacking. Our study is the first large study to compare this 72 h strategy with the policy of continuing antibiotic therapy until neutrophil recovery.

**Implications of all the available evidence**

The results of this large retrospective cohort study suggest that in AML and MDS patients undergoing remission induction chemotherapy it is safe to stop antibiotics after 3 days of FN when there is no clinically or microbiologically documented infection. This study provides further evidence that early discontinuation of antibiotic treatment is safe. This insight could have a significant impact on the use of empirical broad-spectrum antibiotic treatment in neutropenic patients as well as antimicrobial stewardship.

Alt-text: Unlabelled box

In 2017, the How Long study was published [4]. This randomized controlled trial strongly suggests that the early discontinuation of antibiotics given for FN is safe when the patient has become afebrile for 72 h after the start of EBAT. Compared to the standard end-of-neutropenia approach, this clinically oriented approach significantly increased the number of antibiotic-free days (i.e. the primary endpoint), without adversely affecting mortality or days of fever (i.e. the secondary endpoints). In 2009, a prospective observational study at Erasmus MC University Medical Center in Rotterdam, The Netherlands, showed that in 166 patients with AML, the use of a FN protocol which advocates the discontinuation of antibiotic therapy after 72 h when no infection is diagnosed is safe even in patients with ongoing fever [5]. Though promising, this single center non-randomized study cannot be considered sufficient evidence to change treatment guidelines. We performed a large retrospective comparative cohort study to assess if the "3-day EBAT strategy" is safe in AML patients with FN during their first induction chemotherapy. We focused on the first remission induction chemotherapy episode in order to have a study population as homogeneous as possible.

## 2 Methods

### 2.1 Design

**Fig. 1:**
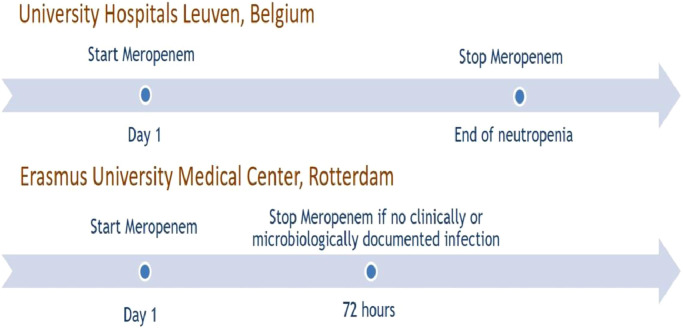
the different strategies in both centers for empirical antibiotic treatment.

The ‘Stopping Antibiotic therapy after 72 h in patients with FEbrile neutropenia undergoing intensive chemotherapy for AML/MDS study’, that we gave the acronym SAFE, is a retrospective multicenter comparative cohort study, performed at the Erasmus MC University Medical Center (Erasmus MC) in Rotterdam in the Netherlands, and at the University Hospitals Leuven (UZL) in Belgium. Both hospitals have large tertiary-care hematology units and treat patients with AML/MDS with the same chemotherapy protocols as part of the HOVON network. Both also use antimicrobial prophylaxis with a fluoroquinolone and fluconazol. In Erasmus MC, it is standard practice to administer three days of broad-spectrum antibiotics (meropenem in this case) for a FN episode, and stop if no microbiologically or clinically documented infection was found as the likely cause of the fever, even when fever has not yet resolved. In this study, we referred to this practice as short course EBAT. At UZL, meropenem is continued for at least seven days and often until neutropenia has resolved, following international guidelines (Fig. 1). As the only difference is situated in the approach towards the treatment of patients with FN, a unique opportunity was provided to compare both strategies within this study.

Fever was defined as one single measured tympanic membrane temperature of > 38.5 °C or a temperature of > 38·0 °C during 2 subsequent measurements separated by at least 2 h. Neutropenia was defined as an absolute neutrophil count (ANC) below 0·5 × 10^9^ cells/L expected to last longer than 7 days.

### 2.2 Inclusion

A list of patients was retrieved via the pharmacy that received induction chemotherapy in both centres. Only patients of 18 years of age or above with a newly diagnosed acute myeloid leukemia (AML) or high-risk myelodysplastic syndrome (MDS), receiving their first induction chemotherapy (3 + 7 regimen) between January 8, 2011 and July 25, 2019 were included. Exclusion criteria were age under 18 years and a history of intensive chemotherapy for AML/MDS or a hematopoietic stem cell transplantation. The study was approved by the Ethics Committee Research of both centers. Because retrospective nature of the study the need for an informed consent was waived.

### 2.3 Endpoints

The primary endpoint was the occurrence of a Serious Medical Complication (SMC) within 30 days after the start of induction chemotherapy. An SMC was defined as either death or admission to the ICU. We chose this objective endpoint because our study was unblinded and an SMC is the most clinically relevant endpoint and the greatest fear for clinicians when stopping EBAT early.

Secondary outcome variables were all-cause mortality within 30 days and within 3 months after the start of the first induction chemotherapy, infection related mortality within 30 days, total duration of any antibiotic treatment (excluding prophylaxis), duration of broad-spectrum antibiotic treatment (i.e. meropenem), duration of gram-positive antibiotic treatment (i.e. glycopeptides or linezolid), the number of days with fever, incidence and types of clinically defined infections, incidence of bacteremia, the highest qSOFA-score for patients with bacteremia, and the type of microorganisms detected in blood cultures. To conclude, we calculated a number of outcome variables specifically for the group that received a short-course EBAT. Definitions are provided in the supplementary materials.

### 2.4 Statistical analysis

Differences between continuous variables (e.g. duration of antibiotic treatment, days of fever, etc.) were assessed with the Mann-Whitney U test. Differences for categorical variables (bacteremia, qSOFA scores) were assessed using a χ² test or Fishers exact test. For the analysis of the primary endpoint of SMC, a multivariable Cox regression model was used, adjusted for the following covariates: age, HCT-CI score, European Leukemia Network AML risk stratification and year of admission. For the analysis of all-cause mortality, a multivariable Cox regression was used and adjusted for the same set of covariates. SPSS version 25 (IBM, Armonk, NY, USA) was used for the analyses. The results for the univariate analyses presented on Table 1 were adjusted for multiplicity using the Holm (or Bonferroni) method. For all tests a significance level of 0·05 was used. A statistician from the department of Biostatistics at Erasmus MC (GP) supervised the statistical analysis.

**Table 1.** Patient characteristics. For age and HCT-CI score, the median with interquartile ranges are reported.

	EMC(*n* = 305)	UZL(*n* = 270)	*p* value
**Sex**			1·000
**Male**	177 (58%)	150 (55·6%)	
**Female**	128 (42%)	120 (44.4%)	
**Age (years)**	62 (53–69)	61 (49–66)	**0**·**024**
**HCT-CI**	2 (1–4)	3 (2–4)	**<0**·**001**
**HCT-CI (excl pulmonary)**	1 (0–3)	1 (0–3)	0·248
**Cardiovascular disease**	41 (13·4%)	44 (16·3%)	1·000
**Diabetes Mellitus**	26 (8·5%)	18 (6·7%)	1·000
**Obesity (BMI>**30 kg**/m2)**	16 (5·2%)	17 (6·3%)	1·000
**Moderate/severe hepatic disease**	32 (10·5%)	7 (2·6%)	**0**·**002**
**Moderate/severe renal disease**	11 (3·6%)	4 (1·5%)	0.884
**Infection at admission**	71 (23·3%)	36 (13·3%)	**0**·**024**
**Colonization by a resistant pathogen**	15 (4·9%)	9 (3·3%)	1.000
**AML Type**			1.000
**Favorable**	81 (26.6%)	60 (22.2%)	
**Intermediate**	72 (23.6%)	82 (30.4%)	
**Adverse**	124 (40.7%)	99 (36.7%)	
**Unclassifiable**	28 (9.2%)	29 (10.7%)	
**Year of admission** 2011–2013 2014–2016 2017–2019	0 161 (52·8%) 144 (42·7%)	102 (37·8%) 80 (29·6%) 88 (32·6%)	**<** **0**·**001**
MDS	27 (8.9%)	17 (6.3%)	1.000

With a power of 90% and a non-inferiority margin of 10% a sample size of 268 patients per arm was calculated to detect non-inferiority in safety of short EBAT treatment versus extended treatment. The serious medical complication rate was assumed to be 15% for both arms based on literature and local data. The sample size calculation was based on a two-sample non-inferiority test for proportions using a significance level of 2.5%.

### 2.5 Role of the funding source

There was no funding source for this study. The authors had full access to the database and made the decision to submit for publication.

## 3 Results

### 3.1 Participants and baseline characteristics

**Fig. 2:**
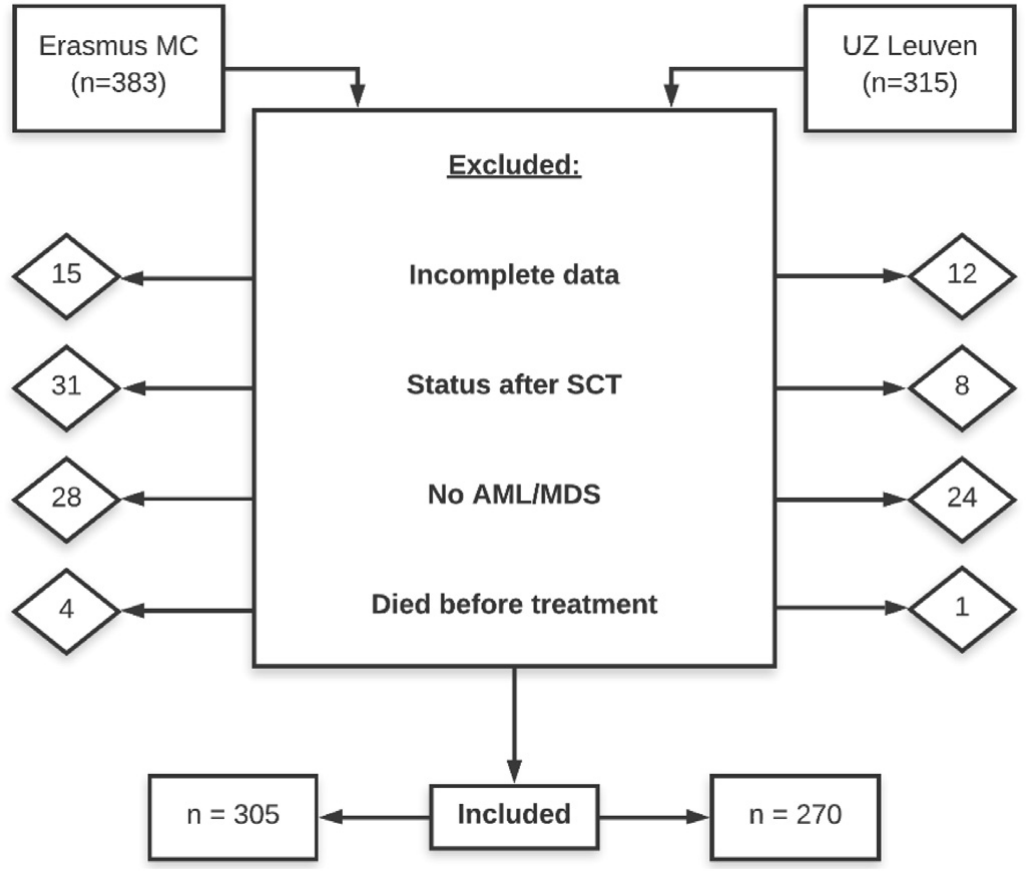
inclusion and exclusion of patients.

A total of 698 patients were screened for eligibility. As shown in Fig. 2, 123 patients were excluded for the following reasons**:** incomplete data, status after stem cell transplantation (SCT), other hematologic malignancy than AML/MDS and death before the start of induction chemotherapy. The total study population consists of 305 patients from the Erasmus MC and 270 patients from University Hospitals Leuven (UZL).

Baseline patient characteristics are summarized in Table 1. In Erasmus MC 8·9% of patients had MDS versus 6·3% in UZL (p = 1·000). The HCT-CI (Hematopoietic Cell Transplantation-Comorbidity Index) is a score for the burden of comorbidities calculated based on 17 different categories of organ dysfunction [6]. The HCT-CI score excluding the pulmonary values was calculated separately because at Erasmus MC a spirometry was not routinely performed at admission, in contrast to UZL.

### 3.2 Serious medical complications (primary endpoint)

Thirty-eight of the 305 patients at Erasmus MC (12·5%) experienced an SMC, compared to 24 out of 270 UZL patients (8·9%), as shown in *Table S1*. At Erasmus MC and UZL, the 30-day ICU admission rates were 9·2% (28 patients) and 7% (19 patients) respectively while the day 30 overall mortality was 8·5% and 4·4%. There was no difference in terms of underlying disease (AML versus MDL).

A Cox regression was performed to compare the number of SMCs between both centers, adjusted for age, AML risk, non-pulmonary HCT-CI score and year of admission. Adjusted for confounding factors, there was no significant difference between both centers in the number of SMCs, with a hazard ratio (HR) of 1·357 (95% CI [0·765 – 2·409]) (*p* = 0·297). As expected, there was a significant effect of the baseline non-pulmonary HCT-CI score, as well as age on the HR for an SMC. There was no significant effect for year of admission and AML risk classification. (See supplementary materials: *Table S2 and Fig. S3*)

### 3.3 Mortality analysis

The all-cause mortality at 30 days after the start of induction chemotherapy was 8·5% (*n* = 26) at EMC versus 4·4% (*n* = 12) at UZL (*p* = 0.049). Ninety-day mortality was 17% (*n* = 52) at EMC, compared to 12% (*n* = 32) of UZL patients (*p* = 0·078).

A multivariate cox-regression for 90-day survival was also performed, using the same covariates as for the analysis of SMCs, as shown in *Table S4 and Fig. S5.* There was no significant difference observed between both centers (HR 1·368, 95% CI [0·848–2·209]). Finally, there was no significant difference in the Cox regression for survival of the first 30 days after the start of chemotherapy and in the univariate analysis for patients in whom death was considered infection related (3.6% [*n* = 11] at EMC versus 1·9% [*n* = 5] at UZL, *p* = 0·310).

### 3.4 Duration of antibiotic treatment in both groups

The median duration of broad-spectrum antibiotic treatment was 9 days (IQR 5–13) at Erasmus MC, versus 19 days (IQR 13–25) at UZL (*p* <0·001). For patients who received treatment with antibiotics against gram-positive bacteria (glycopeptide or linezolid), the median duration was 9 days (IQR 6–13) at Erasmus MC, compared to 11 days (IQR 7–17) at UZL (*p* = 0**.**010). At Erasmus MC, 221 patients (72·5%) received a short-course of EBAT during their first episode of febrile neutropenia, compared to 25 patients (9**·**3%) at UZL (*p* < 0**·**001). Of this population, 103 patients at Erasmus MC and 17 patients at UZL switched to a smaller spectrum antibiotic therapy.

### 3.5 Incidence and types of infection

At EMC 99 patients (32**·**5%) developed a bacteremia during the study period, compared to 73 patients (27%) at UZL (*p* = 0·156). The highest qSOFA score of these patients was calculated and showed no significant difference (*p* = 0·424). Blood culture results are listed in Table 2. As expected, the majority of cultured bacteria were gram-positive in both centers, although a higher incidence of *staphylococcal* bacteremia was observed at Erasmus MC while bacteremia with enterococci was more frequent at UZL. In Erasmus MC, 2 patients were diagnosed with Clostridium difficile infection, versus 4 in UZL (0·7% vs 1·5% respectively, *p* = 0·427). The number of patients that experienced a clinically defined infection (CDI) at least once is shown in *Fig. S7.*

**Table 2.** (Upper) number of patients experiencing bacteremia and types of bacteremia found in their blood cultures. (below) number of patients with candidemia. When more than 1 microorganism was detected in the same patients in one or more blood culture (e.g. gram-positive -and negative bacteria), both were documented. However, when different bacteria were classified in the same category, they were only counted once for that category (e.g. staphylococcus epidermidis and hominis). CNS = coagulase-negative staphyloccus.

	EMC (*n* = 99)	UZL (*n* = 73)	*p*-value
**Gram-positive**	97 (98%)	67 (91·8%)	0·338
**Staphylococcus**	80 (80·8%)	47 (64·4%)	0·139
**CNS**	77 (77·8%)	46 (63%)	0·238
**S. Aureus**	3 (3%)	1 (1·4%)	1·000
**Streptococcus**	11 (11·1%)	5 (6·8%)	1·000
**Enterococcus**	13 (13·1%)	21 (28·8%)	0·109
**Other gram-positives**	9 (9·1%)	6 (8·2%)	1·000
**Gram-negative**	10 (10·1%)	11 (15·1%)	1·000
**Candidemia**	9 (3%) of all patients	1 (0·4%) of all patients	0·181

### 3.6 Duration of fever

295 patients at Erasmus MC (96·7%) and 235 at UZL (87%) developed fever (*p* < 0·001) during the entire follow up. The median number of days of fever was higher at Erasmus MC than at UZL (8 versus 6 days, *p* = 0·015).

### 3.7 Patients treated with short course EBAT at erasmus MC

Of the 305 Erasmus MC patients, 221 patients received a short-course of EBAT in response to their first episode of fever. In 79 of these cases (36·7%), EBAT was stopped, even though the patient still had fever. Following this first course of EBAT, 112 patients (50·7%) received a subsequent course of EBAT due to another fever episode. Thirteen of the 221 patients (5·9%) were admitted to the ICU within 30 days after start of the induction chemotherapy and 17 (7·7%) died. Broad-spectrum antibiotics had not yet been stopped at the time of death in 12 of these 17 cases.

## 4 Discussion

This study assessed and compared the safety of 2 febrile neutropenia treatment strategies in patients with AML or MDS undergoing remission induction chemotherapy. The results of this large retrospective comparative cohort study strongly suggest that the short-course EBAT strategy does not result in a higher risk for an SMC compared to a strategy where EBAT is continued until neutrophil recovery. Also, the number of patients that died in the 30 days after the start of chemotherapy and in whom death was considered infection related was low in both groups. Compared with the long-term EBAT strategy, the short-course strategy very significantly reduced the days that patients were receiving EBAT.

Recently, the How Long Trial demonstrated the safety of stopping empirical EBAT when neutropenic patients have been afebrile and hemodynamically stable for 72 h, compared to the continuation of EBAT until recovery from neutropenia [4]. Also, a small prospective observational study described the apparent safety of stopping EBAT on day 5 regardless of fever or neutropenia resolution [7]. The approach we describe here confirms these observations while going one step further. Indeed, it strongly suggests that EBAT can already be discontinued safely after 72 h and regardless of the resolution of fever as long as no infection was documented clinically or microbiologically. Compared with a strategy where EBAT is continued until neutrophil recovery, the 72 h approach led to a substantial 10-day decrease in the use of broad-spectrum antibiotics. This increase in antibiotic-free days is substantially higher than the 2·4 days observed in the How Long Trial [4].

It is clear that a reduction in the number of days on EBAT has several advantages. It reduces the risk for colonization (and subsequent infection) with resistant gram-negative and positive bacteria [8,9]. Secondly, antibiotic therapy is not without direct or indirect side-effects, which occasionally can be life-threatening (e.g. pseudomembranous colitis) [10]. Finally, broad-spectrum antibiotic therapy impacts the gut microbiome, which has been associated with a poorer outcome of hematopoietic stem cell transplantations due to a higher risk of graft-versus-host-disease [11]. Furthermore, longer exposure to EBAT can cause selection of certain bacterial taxa in the microbiome (mostly enterococci), to the disadvantage of some protective clostridiales. This dysbiosis might increase the risk of bacterial translocation and bacteremia [12,13].

In our secondary analyses, we investigated the occurrence of different CDIs and MDIs. No difference in the overall incidence of bacteremia was observed. However, differences in the type of gram-positive bacteremia were observed with staphylococcal species being more frequent at Erasmus MC, while enterococcal bacteremia was more frequent at UZL. This may be explained by the extended use of meropenem, as well as the use of vancomycin-locks as adjunctive therapy in patients with gram-positive bacteremia at UZL, a therapy not used at Erasmus MC. Finally, at Erasmus MC, colistin is administered orally during the first 10 days after the start of chemotherapy in order to accelerate the eradication of gram-negatives and to minimize the risk of developing quinolone resistance. This approach could select for gram-positive bacteria and increase the incidence of gram-positive bacteremia.

Our study has its limitations. Its retrospective design makes it impossible to rule out any remaining confounders, even though the two centers were comparable regarding the patient population (all AML or MDS undergoing first remission induction chemotherapy), the use of meropenem as EBAT, and the use of fluconazole and quinolone prophylaxis. However, as explained above, differences were present regarding the use of colistin prophylaxis and vancomycin-lock therapy. Because of the retrospective design of the study causality cannot be ascertained. Also, it is difficult to assess if the criteria used by the clinician to decide whether there is a microbiologically or clinically documented infection are uniform. Therefore, given the retrospective design, the data on the incidence of clinically defined infections should be interpreted with caution.

To the best of our knowledge, this study is the largest comparative study on early versus late discontinuation of antibiotic therapy for FN in AML/MDS patients treated with induction chemotherapy. Other studies published on the subject have included patients with different hematologic malignancies and thus different durations of neutropenic episodes. Previous studies that were conducted either lacked a control group, included a more heterogeneous population, or studied a different policy. Furthermore, in contrast to the How Long Trial which focused on antibiotic free days, we focused on serious medical complications as the primary endpoint, which we think is a more relevant endpoint [4].

In a recent meta-analysis, Stern et al. assessed the safety of discontinuation of antibiotics regardless of neutrophil count, compared to the continuation until neutropenia resolution, in cancer patients [14]. In total, data from 662 distinct febrile neutropenia episodes were included from eight clinical trials, all from before the year 2000. No significant difference in all-cause mortality between the short- and long antibiotic therapy arms was observed (RR 1·38, 95%; CI 0·73–2·62). Furthermore, they did not find significant differences between both groups in the incidence of bacteremia.

The insights that our study provides alongside the already existing evidence could help to change a decades-long practice and could further improve hospital-based antimicrobial stewardship programs [15]. However, an adequately powered randomized controlled trial would be needed to further prove the safety of the short-course EBAT strategy and have this strategy advocated by international guidelines.

## Funding

There was no specific funding for this study.

## Data sharing statement

The deidentified data that support the findings of this study are available upon reasonable request from the corresponding author AS, beginning 9 months and ending 36 months following article publication.

## Contributors

Each author has made substantial contributions to the conception or acquisition of the study, to the analysis or interpretation of data used in the work or has drafted the work or substantively revised it. Each author has approved the submitted version and agrees to be personally accountable for the author's own contributions and for ensuring that questions related to the accuracy or integrity of any part of the work are appropriately investigated, resolved, and documented in the literature.

AS, RA, ES, AV, TM and AD drafted the manuscript. AV, ES, RA, AD, BS and TM collected the data. AS, RA, ES, AV, TM, GP, JM, BR and AD analyzed the data. ES, AV, RA, AD and GP run the statistical analysis and designed the table and figures. All authors critically revised the manuscript for important intellectual content and gave final approval for the version to be published.

## Declaration of Competing Interest

AS received non-financial support from Gilead Sciences and Pfizer, outside the context of this work. JM reports grants, personal fees and non-financial support from Pfizer, grants, personal fees and non-financial support from Gilead,personal fees and non-financial support from MSD,personal fees and non-financial support from Cidara, personal fees and non-financial support from F2G,personal fees and non-financial support from Shire/Takeda,personal fees and non-financial support from Scynexis, personal fees and non-financial support from Amplyx, outside the submitted work. TM reports personal fees and non-financial support from Pfizer, grants, personal fees and non-financial support from Gilead Scienses, personal fees from Celgene, non-financial support from MSD/Merck, non-financial support from OLM Diagnostics, non-financial support from IMMY, non-financial support from PathoNostics, non-financial support from FUJIFILM Wako, outside the submitted work. All other authors have no conflicts to declare.

## Appendix Supplementary materials

Image, application 1

Image, application 2
