# Supplementary material for: Stopping antibiotic therapy after 72 h in patients with febrile neutropenia following intensive chemotherapy for AML/MDS (safe study): A retrospective comparative cohort study
Source: eClinicalMedicine. 2021 Apr 25;35:100855. doi: 10.1016/j.eclinm.2021.100855 (PMC8099620; doi:10.1016/j.eclinm.2021.100855)
Supplement: Supplementary file 1 [file mmc1.docx]

# Online Supplementary material

**METHODS**

Antibiotic prophylaxis

All patients in this study received antibacterial prophylaxis with fluoroquinolones (in Erasmus MC ciprofloxacine 500mg BID and in UZL Levofloxacin 500mg OD). In both centers patients received antifungal prophylaxis with fluconazole 400mg OD. Antibacterial and antifungal prophylaxis was started 1 day before or on the day chemotherapy was started and continued until neutrophil recovery. At Erasmus MC, colistin capsules 200 mg QID combined with 5 mg oral colistin suspension QID are administered for the first 10 days of neutropenia as well. Prophylaxis was only given parenterally when oral intake was impaired (e.g. severe mucositis).[5] In both centers no antimould prophylaxis is given and a diagnostic driven approach is performed for fungal infections in both centers. When a patient has persistent fever despite broad-spectrum antibiotics for 96 hours, a chest CT scan is performed and if necessary, subsequently followed by broncho-alveolar lavage sampling for culture and galactomannan testing.

Inclusion and definitions

A list of patients who received intensive chemotherapy (3 days of one of the anthracyclines daunorubicin or idarubicin plus 7 days of cytarabine (3+7 regimen)) between 2011 and 2019 was obtained from the pharmacy department of both hospitals. All patients’ files were analyzed to assess their eligibility for inclusion. Demographic, microbiological and clinical data were extracted manually. The end of study date was defined as the date of discharge home with no unforeseen readmission, date of death (if before the 2nd course of chemotherapy) or the start date of the 2nd course of chemotherapy if the patient was not discharged in between.

For each patient, every antibiotic treatment episode (i.e. a period of uninterrupted days with any antibiotic treatment except prophylaxis) was recorded and for each episode data were collected regarding the type of antibiotic, clinically documented infections (i.e. episodes of clinically defined infection, without microbiological documentation), microbiologically documented infections (i.e. a positive culture from blood, urine, sputum, broncho-alveolar lavage or any other normally sterile body site), the presence of bacteremia (i.e. at least two positive blood cultures containing the same micro-organism), the highest quick Sepsis Related Organ Failure Assessment (qSOFA)-score measured for every patient (a score used for assessing the risk of in-hospital mortality based on respiratory rate, mental status and blood pressure). Additionally, a short course antibiotic treatment was defined as patients receiving broad-spectrum antibiotics for 72 hours or less. The number of days of antibiotic treatment was counted as the number of calendar days on which patients received antibiotics. Subsequently, all febrile episodes during the study period were documented per patient. A fever episode was defined as a tympanic temperature of >38·5°C once or 2 consecutive measures of >38·0°C with at least two hours in between. If a patient had a temperature <38·0°C for at least 24 hours, the fever episode was considered to have ended. Other data collected were the duration of neutropenia, admission to the intensive care unit (ICU), date and cause of death, antifungal treatment that was administered and the criteria for invasive fungal disease as per EORTC classification. We also assessed if patients were colonized by a highly-resistant micro-organism (HRMO).[16] Finally, the Hematopoietic Cell Transplantation-Comorbidity Index (HCT-CI) at the start of chemotherapy was calculated for each patient, as a measure of comorbidities and predictor for mortality. The HCT-CI is an index for the burden of comorbidities calculated based on 17 different categories of organ dysfunction. The HCT-CI does not only predict non-relapse mortality after allogeneic stem cell transplantation but correlates well with mortality in patients with AML receiving induction chemotherapy.[6] To correct for the fact that all patients received a spirometry preceding the start of chemotherapy at UZL, but not so at Erasmus MC, the HCT-CI score without pulmonary values was also calculated.

**RESULTS**

Serious medical complications

**Table S1: Distribution of SMCs and its components in both centers.**

|  | **EMC** | **UZL** | **Univariate p-value** |
| --- | --- | --- | --- |
| **Death within 30 days** | 26 (8·5%) | 12 (4·4%) | 0·049 |
| **ICU admissions within 30 days** | 28 (9·2%) | 19 (7%) | 0·27 |
| **Total SMCs** | 38 (12·5%) | 24 (8·9%) | 0·17 |
| **Male** | 23 | 14 | 0·30 |
| **Female** | 15 | 10 | 0·38 |

**Table S2: Hazard ratios for experiencing an SMC in Erasmus MC compared to UZL, adjusted for age at inclusion, AML risk, HCT-CI score (excluding pulmonary values) and year of admission.**

|  | Hazard ratio for an SMC (95% CI) |
| --- | --- |
| **Medical center** | 1.458 (0.802 – 2.652) |
| **Age at inclusion** | 1.025 (0.999 – 1.050) |
| **AML risk classification** | 1  0.779 (0.374 – 1.621)  0.654 (0.331 – 1.295)  0.940 (0.363 – 2.434) |
| **Favorable**  **Intermediate**  **Adverse**  **Unclassifiable** |  |
| **HCT-CI without pulmonary score** | 1.194 (1.042 – 1.369) |
| **Year of admission** | 0.935 (0.820 – 1.066) |


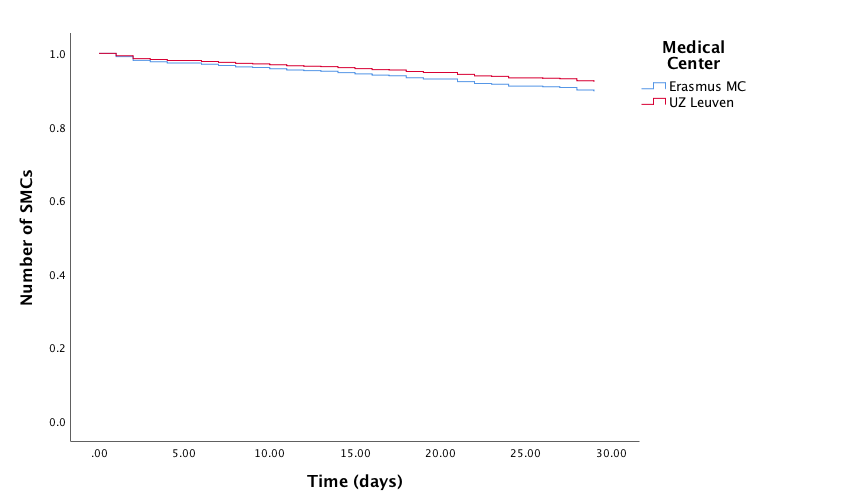


**Figure S3: Cox regression of the number of SMCs in both centers, adjusted for age, non-pulmonary HCT-CI score and year of admission.**

Mortality

**Table S4: Hazard ratios for death at 90 days in Erasmus MC compared to UZL, adjusted for age at inclusion, AML risk, HCT-CI score (excluding pulmonary values) and year of admission.**

|  | Hazard ratio for Death at 90 days (95% CI) |
| --- | --- |
| **Medical center** | 1.420 (0.830 – 2.430) |
| **Age at inclusion** | 1.045 (1.019 – 1.073) |
| **AML risk classification** | 1  1.234 (0.593 – 2.568)  1.467 (0.758 – 2.841)  1.445 (0.571 – 3.661) |
| **Favorable**  **Intermediate**  **Adverse**  **Unclassifiable** |  |
| **HCT-CI without pulmonary score** | 1.348 (1.191 – 1.525) |
| **Year of admission** | 0.958 (0.851 – 1.079) |

**
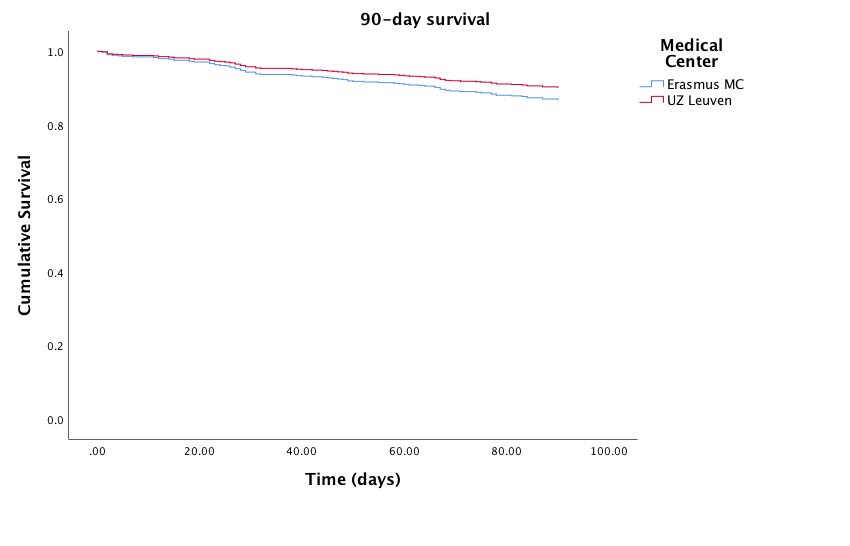
**

**Figure S5. Cox regression of death after 90 days in both centers, adjusted for age, AML risk, non-pulmonary HCT-CI score and year of admission.**

|  | **EMC**  n = 305 | **UZL**  n = 270 | **p-value** |  |
| --- | --- | --- | --- | --- |
| **No fungal pneumonia** | 263 (86.2%) | 254 (94.1%) | 0.021 | |
| **Possible fungal pneumonia** | 42 (13.8%) | 16 (5.9%) |  | |
| **Proven** | 0 (0%) | 0 (0%) |  | |
| **Probable** | 17 (5.6%) | 10 (3.7%) |  | |
| **Possible** | 25 (8.2%) | 6 (2.2%) |  | |

**Table S6. Incidence of fungal pneumonia categorized according to the EOTRC classification.**


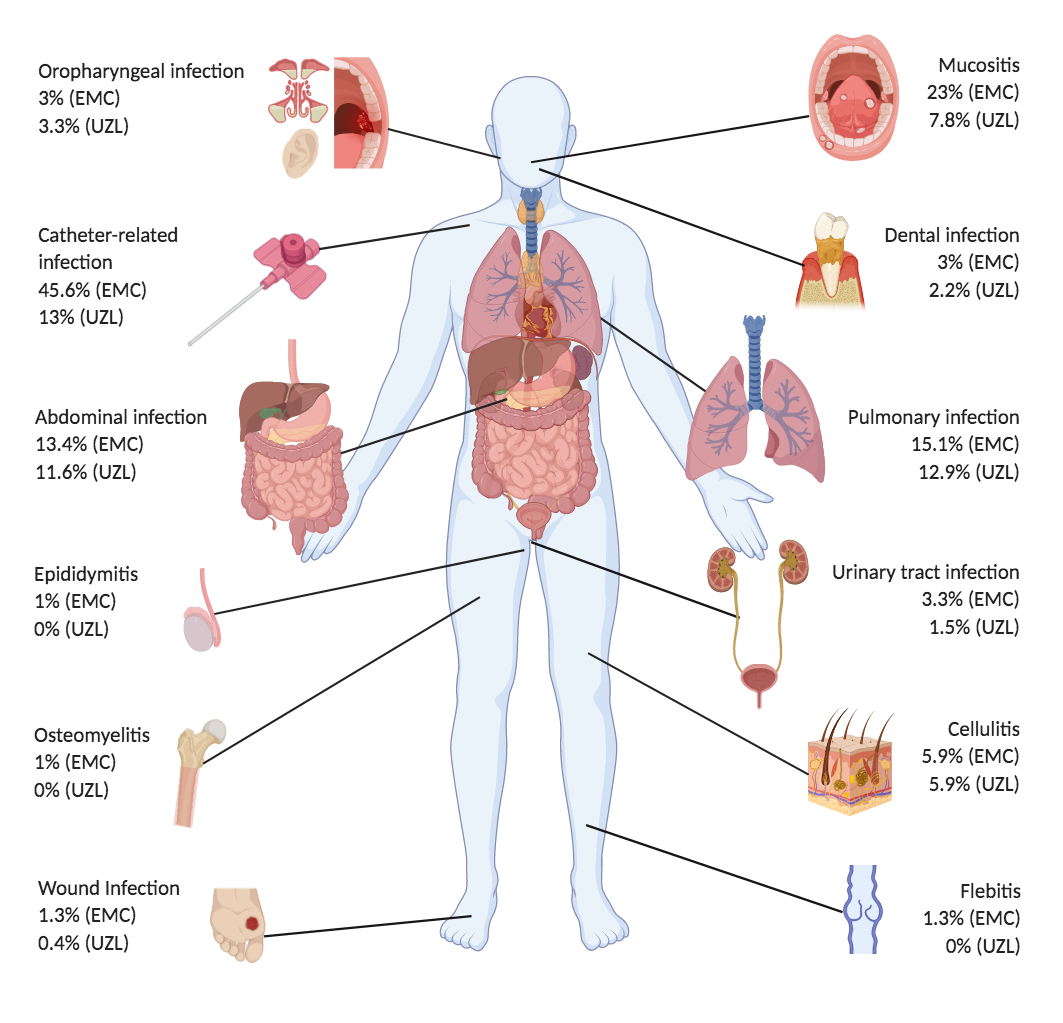
Different CDIs

**Figure S7: The occurrence of different CDIs. If proportions were <1%, the infection was not visualized.**

**Table S8: Hazard ratios for experiencing an SMC in Erasmus MC compared to UZL, adjusted for age at inclusion, MDS, HCT-CI score (excluding pulmonary values) and year of admission.**

|  | Hazard ratio for an SMC (95% CI) |
| --- | --- |
| **Medical center** | 1.427 (0.779 – 2.616) |
| **Age at inclusion** | 1.033 (1.006 – 1.061) |
| **MDS** | 0.844 (0.287 – 2.481) |
| **HCT-CI without pulmonary score** | 1.168 (1.016 – 1.342) |
| **Year of admission** | 0.936 (0.819 – 1.070) |
