# Supplementary material for: Stopping antibiotic therapy after 72 h in patients with febrile neutropenia following intensive chemotherapy for AML/MDS (safe study): A retrospective comparative cohort study
Source: eClinicalMedicine. 2021 Apr 25;35:100855. doi: 10.1016/j.eclinm.2021.100855 (PMC8099620; doi:10.1016/j.eclinm.2021.100855)
Supplement: Supplementary file 2 [file mmc2.docx]

# Caption for supplementary material

- METHODS
  - Antibiotic prophylaxis
  - Inclusion and definitions
- RESULTS
  - Table S1: Distribution of SMCs and its components in both centers.
  - Table S2: Hazard ratios for experiencing an SMC in Erasmus MC compared to UZL, adjusted for age at inclusion, AML risk, HCT-CI score (excluding pulmonary values) and year of admission.
  - Figure S3: Cox regression of the number of SMCs in both centers, adjusted for age, non-pulmonary HCT-CI score and year of admission.
  - Table S4: Hazard ratios for death at 90 days in Erasmus MC compared to UZL, adjusted for age at inclusion, AML risk, HCT-CI score (excluding pulmonary values) and year of admission.
  - Figure S5. Cox regression of death after 90 days in both centers, adjusted for age, AML risk, non-pulmonary HCT-CI score and year of admission.
  - Table S6. Incidence of fungal pneumonia categorized according to the EOTRC classification.
  - Figure S7: The occurrence of different CDIs. If proportions were <1%, the infection was not visualized.
  - Table S8: Hazard ratios for experiencing an SMC in Erasmus MC compared to UZL, adjusted for age at inclusion, MDS, HCT-CI score (excluding pulmonary values) and year of admission.
